# Assessing the links between childhood trauma, C-reactive protein and response to antidepressant treatment in patients with affective disorders

**DOI:** 10.1007/s00406-021-01245-z

**Published:** 2021-03-17

**Authors:** Kai F. Fischer, Maria S. Simon, Julie Elsner, Johanna Dobmeier, Johannes Dorr, Leonie Blei, Peter Zill, Michael Obermeier, Richard Musil

**Affiliations:** 1 Department of Psychiatry and Psychotherapy, University Hospital, LMU Munich, Nußbaumstraße 7, 80336 München, Germany; 2 GKM Gesellschaft für Therapieforschung mbH, Munich, Germany; 3 Institute of Mental Health at UBC, University of British Columbia, Vancouver, Canada; 4 Endopraxis Amberg, Amberg, Germany; 5 Clinic for Internal Medicine South, Munich, Germany

**Keywords:** Childhood trauma, Inflammation, CRP, Depressive disorder, Treatment resistance

## Abstract

Adverse Childhood Experiences (ACE) are a well-known risk-factor for depression. Additionally, (high-sensitive) C-reactive Protein (hsCRP) is elevated in subgroups of depressed patients and high following ACE. In this context the literature considers hsCRP and ACE to be associated with treatment resistant depression. With the data being heterogenous, this study aimed to explore the associations of ACE, hsCRP levels and response to antidepressant treatment in uni- and bipolar depression. *N* = 76 patients diagnosed with uni- or bipolar depression and *N* = 53 healthy controls were included. Treatment was over 6 weeks in an inpatient psychiatric setting within an observatory study design. Depressive symptoms were assessed by the Montgomery-Asberg Depression Rating Scale (MADRS), ACE were assessed by the Childhood Trauma Questionnaire (CTQ); the body-mass-index (BMI) and hsCRP were measured. HsCRP levels did not differ between the study population and the healthy controls. While the depressive symptoms decreased, the hsCRP levels increased. Sexual abuse was associated with significant higher and emotional abuse with lower levels of hsCRP after 6 weeks. The baseline hsCRP levels and the ACE subgroups did not show significant associations with the treatment response in unipolar depressed patients. The long-lasting effects of specific forms of ACE may have relevant impact on inflammation, supporting hsCRP to be a suitable biomarker. With ACE and hsCRP not showing any significant associations with treatment response in the unipolar depressed subgroup, a more differentiate research concerning biomarkers and treatment regimens is needed when talking about treatment response.

## Introduction

Early life stress is among the most important risk factors for physical and mental health problems [2, 22, 24, 39, 53, 60]. Adverse Childhood Experiences (ACE), such as emotional abuse and neglect, physical abuse and neglect and sexual abuse, are more frequent in numerous psychiatric diseases [2, 3, 13, 26, 53]. ACE are furthermore associated with a worse clinical course [20, 68], higher rates of suicide attempts [2], recurrent episodes [58] and treatment resistance [77] within uni- and bipolar depression.

But also chronic somatic diseases like Diabetes mellitus or coronary heart disease, who are amongst the leading causes of death [4, 33, 41, 53] are all more often after ACE. Evident underlying pathogenetic mechanisms are increased incidences of traditional risk factors such as smoking, over-eating or alcohol abuse (e.g., [21, 72]) used as dysfunctional coping strategies to regulate aversive emotions [53]. Furthermore, literature suggests ACE to be a risk factor themselves by causing structural and functional brain alterations (e.g., [62, 82]) as well as a chronic state of inflammation induced by long-lasting psychological stress [23].

In the early stages of life, human sensitivity to stress is particularly high. Enduring toxic stress exceeds the child’s abilities to cope and leads to a dysregulation of the central biological stress pathway, the hypothalamus–pituitary–adrenal (HPA) axis and an alteration of the human’s autonomic nerval system and immune system [61]. Over time this may cause epigenetic changes in stress-response-genes such as FKBP5 [83] or the monoamine-oxidase-gene [10]. Besides a low heritability, gene-environment interactions through epigenetic alterations are suspected of carrying on the effects of ACE up to adulthood (for an overview see Ref. [38]). As a possible result, people with ACE often find themselves in a chronic state of inflammation [15]. In this context, (high-sensitive) C-reactive protein (hsCRP) is one potential and promising biomarker [7, 17, 18]. Beside the established elevation in obese people [14], recent literature identified higher levels of hsCRP in mental ill and healthy people with a history of ACE [7, 15, 18, 48, 63]. However, when differentiating ACE into single forms of maltreatment, current literature is still heterogenous. While Baumeister et al. [7] and Moraes et al. [56] highlight the relevance of sexual and physical abuse in consecutive inflammatory processes, other authors do not differ between single forms of ACE [15, 19].

Along with ACE, inflammation is discussed to play also an important role in the development of uni- and bipolar depression [65, 69, 71, 80]. While underlying mechanisms remain uncertain, literature indicates that inflammation markers, such as IL-6, TNF-α or hsCRP are elevated in subgroups of patients with uni- and bipolar depression and increase the risk for developing a major depression in healthy adults [12, 19, 31, 65, 74, 79–81].

Amongst evolutionary and biological models (for an overview see Ref. [52]), numerous authors support the hypothesis of multifactorial sequential pathways from enduring stress arising from ACE, epigenetic alterations, dysfunctional coping strategies and inflammation to depression [1, 30, 63, 74].

The inflammation hypothesis of depression furthermore suggests elevated inflammation markers to play a key role in the course and response to anti-depressant treatment [12, 71]. Current research focuses on the question whether inflammation markers can predict anti-depressant therapy response with suggesting higher inflammation to be associated with worse response [5, 12, 49]. A recent review of Arteaga-Henriquez et al. [5] stated that the neurotransmitter systems (serotonin, noradrenaline, dopamine) targeted by an anti-depressant drug are crucial for the response. Depressed patients with a low-grade inflammation (hsCRP levels < 1 mg/l) seem to already profit from first-line serotonergic substances, while add-on dopaminergic, noradrenergic and glutamatergic as well as add-on anti-inflammatory medication are associated with better response rates in patients with higher hsCRP levels > 1 mg/l. In this context, data concerning the alteration of the potential biomarkers during a depressive episode and therefore their ability to indicate an amelioration is very heterogenous in current literature [49, 76]. For a better understanding of the underlying mechanisms in the development of depression and the long-lasting effects of ACE, as well as in the search for new treatment strategies in depression the associations of ACE, inflammation and treatment response appear to play an important role.

Therefore, the present study aims to explore first, whether different forms of ACE are associated with altered hsCRP levels, second, if specific forms of ACE are associated with the response to anti-depression treatment, third, whether the treatment response is also associated with hsCRP levels, and fourth, whether the hsCRP levels vary during anti-depressant treatment.

## Methods

### Study design

We analysed data from an observational prospective clinical trial with a naturalistic design conducted in the Department of Psychiatry and Psychotherapy of the University Hospital Munich (Ludwig-Maximilians-Universität München). There were no constraints concerning the applied medication. After inclusion, the drugs prescribed were chosen according to clinical experience and in consideration of the recommendations of the national guideline for uni- and bipolar depression. Besides pharmacological treatment, therapy included a multidisciplinary treatment consisting of ergo- and physiotherapy as well as a regular psychoeducation group based on cognitive-behavioural therapy. The study duration was 6 weeks after admission to the hospital. All procedures were approved by an independent local ethics committee and the study was conducted according to the criteria for Good Clinical Practice as well as to the Declaration of Helsinki in its last revision.

### Study population

*N* = 76 patients who met the diagnostic criteria for uni- or bipolar depression (F31.x, F32.x, F33.x) according to the International Cluster of Differentiation (ICD-10) were included. Further inclusion criteria were an age of 18–65 years and written informed consent. Exclusion criteria were an ongoing substance abuse (except for nicotine), a relevant physical disease, a co-morbid personality disorder (F60.x), a maniac episode (F31.0, F31.1) and a diagnosis out of the schizophrenia spectrum (F20.x).

The healthy control group (*N* = 53) was matched to the study group in terms of age. With a structured interview, the control subjects were screened for a history of or present psychiatric disorder. Depressive symptoms were screened using the Beck Depression Inventory (BDI) [8, 43]. To fulfil inclusion criteria for the healthy control group, an absence of a psychiatric diagnosis and a total BDI score < 10 were necessary.

### Assessments

To assess the severity of depressive symptoms and for a sufficient comparability the Montgomery-Asberg Depression Rating Scale (MADRS) [54, 67], the Hamilton Depression Rating Scale (HAMD) in its 21 item version [28] and the self-measurement questionnaire Beck Depression Inventory (BDI) [43] were applied. All three represent well-validated and established instruments in assessing depressive symptoms.

Concerning the ACE, the Childhood Trauma Questionnaire (CTQ) [78] was used. The CTQ is a 28-item self-report questionnaire assessing five subscales of childhood maltreatment retrospectively: emotional abuse and neglect, physical abuse and neglect and sexual abuse. The subscales are measured on a 5-point Likert scale with the range from 1 (never true) to 5 (very often true). Three minimization or denial items are also part of the questionnaire. For the subscales and the total trauma burden scores can be calculated; it is also possible to make a categorial evaluation with a “exist” or “doesn’t exist” statement for each form of childhood maltreatment. The Questionnaire demonstrates good validity and reliability (cronbach’s alpha 0.95, intra-class correlation 0.88) [9]. In the present study, we used the German version of the CTQ [78]. All ratings were performed by trained rater. At baseline, socio-demographic variables and the CTQ were assessed, BMI (kg/m^2^) was calculated. MADRS-, HAMD- and BDI-scores were rated at baseline, and after 2 and 6 weeks.

For measuring high-sensitive CRP, peripheral blood samples were collected at baseline and additionally after 6 weeks in the study population. Analysis were performed by the Laboratory for Psychiatric Genetic and Neurochemistry in the Department of Psychiatry and Psychotherapy of the University Hospital Munich (Ludwig-Maximillians-Universität München). After centrifugation with 1500*g* for 4 min at 4 °C, hsCRP was assessed in EDTA-plasma with a commercially available ELISA Kit (Human High Sensitivity C-Reactive Protein; hsCRP, Cusabio, Houston, USA). Detection was conducted on the Polarstar Optima Plate Reader (BMG Labtech, Ortenberg, Deutschland).

### Statistics

For identifying group differences between the healthy control group and the study population as well as for differentiating the early-improver/responder subgroup concerning hsCRP and to analyse the course of the depressive symptoms in the unipolar depression subgroup (ΔMADRS, ΔHAMD, ΔBDI), non-parametric tests (Wilcoxon test, Man-Whitney *U* test) were performed.

To analyse the association between ACE and hsCRP, multiple regression analysis were performed under correction of the BMI and smoking state as a confounder of inflammation. For the calculations, hsCRP was log10 transformed due to concerning residual plots for the final models without log transformation.

To analyse the association between ACE, hsCRP and treatment response, multiple regression analysis were performed under correction of the BMI and the state concerning recurrent depression as well know confounder for Inflammation, respectively, treatment response. Due to the ongoing discussion of the use of antidepressants in bipolar disorder the patients with a bipolar depression were excluded from the analyses concerning treatment response.

For the multiple regression analysis, we used the total scores of the MADRS and the subscales of the CTQ. To increase sensitivity, we refrained from including continuous (total number) and dichotomous (exposed vs. not exposed) measures for the childhood adversities. To measure response to antidepressant treatment, *ΔMADRS_1* was calculated by the difference between the MADRS total score at baseline minus the MADRS total score after 2 weeks. For building subgroups, *early improvement* was defined as a 20% reduction of the baseline MADRS Score after 2 weeks. *ΔMADRS_2* was calculated as the difference between MADRS total score at baseline minus the MADRS total score after 6 weeks. At the end of the study period *responder* state was defined as a minimum reduction of 50% of the MADRS total score at baseline.

When the statistical calculations were performed levels of hsCRP > 10 mg/l were excluded. We ran the statistical analysis without probands above this stated threshold to avoid acute infections or physical injury being responsible for elevated inflammation markers. This threshold is recommended by the U. S. Centers for Disease Control and Prevention and the American Heart Association.

To verify the model condition of normal distributed residuals, residual plots of the final models were compiled. All results were corrected for multiple testing via the Bonferroni correction. To ensure a global level of significance of *p* < 0.05, the threshold for each variable in the multiple regression analysis was set at *p* < 0.0071. The statistical analyses were performed with SPSS 25.0 [70].

## Results

Study population and test persons in the healthy control group were matched concerning age and sex. The mean BMI in the study population lay just below the limit of pre-obesity (defined as > 25 kg/m^2^ according to ICD-10) and the majority was diagnosed with a unipolar depression (89.5%). After 6 weeks *N* = 9 patients were excluded from the statistical analyses due to missing data.

Table 1 summarizes the demographic characteristics of the study population and the data of the healthy control group. It also shows the results of the blood samples, Table 2 summarizes the total scores of the questionnaires.

**Table 1:** Demographics of study population and control group

	hsCRP							
	Study population	Healthy controls	Early-improver	Non-early improver		Responder		Non-responder
	Mean (SD)	Mean (SD)	Mean (SD)	Mean (SD)		Mean (SD)		Mean (SD)
*N*	76	53	43	24		37		30
hsCRP baseline (kg/m^2^)	1.63 (2.48)	1.30 (1.46)	1.57 (1.86)	1.96 (3.65)		1.36 (1.67)		2.13 (3.43)
hsCRP 6 weeks (kg/m^2^)	2.67 (3.40)		2.54 (2.50)	2.89 (4.64)		2.12 (2.22)		3.34 (4.40)
sex (m/f)	36/40	26/27	–	–		–		–
Age (years)	43.97 (14.06)	40.20 (16.68)	–	–		–		–
BMI (kg/m^2^)	24.18 (4.52)	–	–	–		–		–
Smoker (%)	37 (48.7)	–	–	–		–		–
Diagnosis (%)	F32/F33: 68 (89.5)			F31: 8 (10.5)				
	F32.2/F33.2	F32.1/F33.1	F33.x/F33.3	F31.3	F31.4		F31	
	37 (48.7)	11 (14.4)	28 (36.8)	3 (3.9)	4 (5.2)		1 (1.3)

**Table 2:** Scales for depression and childhood trauma

Depression				
	MADRS m (SD)	HAMD m (SD)		BDI m (SD)
Baseline	26.00 (7.32)	26.64 (7.02)		46.67 (9.00)
2 weeks	17.46 (9.44)	17.84 (8.69)		39.91 (9.78)
6 weeks	12.90 (7.89)	13.34 (7.71)		30.50 (13.61)
Childhood Trauma Questionnaire				
Subscale	Total score (SD)		Prevalence (%)	
Emotional abuse	9.57 (5.65)		19 (25)^a^	
Emotional neglect	12.12 (5.51)		47 (61.8)^a^	
Physical abuse	6.61 (3.17)		9 (11.8)^a^	
Physical neglect	7.66 (3.5)		16 (21.1)^a^	
Sexual abuse	6.54 (4.21)		10 (13.2)^a^	

^a^Prevalence of the forms of childhood maltreatment equate total scores in the range of *moderate to severe* and *severe to extreme abuse* in the CTQ

The majority of the study population took anti-depressive medication when being admitted to the hospital. Only 11.3% of the probands did not take any psychopharmacologic drugs, whereas almost half of the study population (46.5%) received a combined therapy consisting of a combination of two or more antidepressants or an augmentation of an antidepressant with an atypical antipsychotic drug. 44.7% of the patients took either benzodiazepines or zopiclone when included in the study. Table 3 summarizes the prescribed medication at baseline.

**Table 3:** Medication of the study population at baseline

	Combined therapy + sedatives			Combined therapy − sedatives			Monotherapy SSRI			Untreated	
*N* (%)	47 (66.2)			33 (46.5)			8 (11.3)			8 (11.3)	
		SSRI	SNRI		Mirtazapine	TCA		MAO-I	Sedatives		AAP
*N* (%)		32 (45.1)	10 (14.1)		10 (14.1)	9 (12.7)		6 (8.5)	34 (44.7)		25 (35.2)

*Sedatives* benzodiazepines, zopiclone, *SSRI* selective serotonin reuptake inhibitor, *SNRI* serotonin noradrenaline reuptake inhibitor, *TCA* tricyclic antidepressants, *MAO-I* monoamine oxidase inhibitor, *AAP* atypical antipsychotic, *MS* mood stabilizer

### HsCRP sample characteristics

HsCRP levels in the study population (*m* = 1.63, SD = 2.48) did not differ significantly from the healthy controls (*m* = 1.30, SD = 1.46) at baseline (*U* = 1890.5, *z* = 0.592, *r* = 0.05, *p* = 0.810). Within the subgroups of the study population, neither the *early-improvement* (*U* = 733.5, *z* = 0.340, *r* = 0.05, *p* = 0.734) nor the *responder* state (*U* = 706.5, *z* = 0.840, *r* = 0.05, *p* = 0.401) showed significant differences compared to hsCRP levels in the healthy controls. Concerning the *early improvement* state (early vs. non-early improvement after 2 weeks) in the unipolar depression group, the subgroups did not show significant differences regarding baseline hsCRP (*U* = 523.5, *z* = 0.58, *r* = 0.05, *p* = 0.954). The same applies to the hsCRP levels at the beginning of the study when stratifying the study population into *responder*/*non-responder* state after 6 weeks (*U* = 375.5, *z* = 0.89, *r* = 0.05, *p* = 0.374). At the end of the treatment period, statistical analysis showed a significant increase of hsCRP levels in the overall study population (*m* = 2.67, SD 3.40, *z* = 3.30, *r* = 0.40, *p* = 0.001).

### ACE in the study population

The most frequently reported types of ACE were emotional neglect with 61.8% prevalence rates in the study population, followed by emotional abuse (25%) and physical neglect (21.1%). Sexual and physical abuse were the least frequent but still reported by over 10% of the study population (see Table 2).

### Symptom decrease over treatment time

The total scores of the depression self-questionnaire (BDI, *Z* = 6.847, *r* = 0.05, *p* = 0.000) and the two physician administered questionnaires (MADRS, *Z* = 6.394, *r* = 0.05, *p* = 0.000; HAMD, *Z* = 6.405, *r* = 0.05, *p* = 0.000) significantly decreased during the treatment period. While the mean of the MADRS score indicates moderate depression at baseline, the mean HAMD value equates severe depression. Both questionnaires result in means indicating mild depression after 6 weeks. By contrast, the BDI still reports severe depression at the end of treatment (see Table 2).

### ACE and inflammation

The multiple regression analysis showed no significant associations of the total scores of the CTQ subscales with higher levels of loghsCRP at baseline and after correction for multiple testing. Smoking state showed also no significant associations with the baseline loghsCRP.

When looking at the loghsCRP after 6 weeks sexual abuse (*β* = 0.330, *p* = 0.007) and the BMI (*β* = 0.427, *p* = 0.000) showed significant positive associations. The subscale emotional abuse showed a significant negative correlation with loghsCRP (*β* = − 0.589, *p* = 0.000). Furthermore, the BMI was positively associated with loghsCRP regardless the decrease of the depressive symptoms (see Table 4).

**Table 4:** Multiple regression for loghsCRP with CTQ subscales, BMI and smoking state

	loghsCRP (baseline)						loghsCRP (6 weeks)				
	*β*	Stand *β*	*p*	95% CI			*β*	Stand *β*	*p*	95% Cl	
Emotional abuse	− 0.030	− 0.328	0.049	0.049	− 0.061		− 0.057	− 0.589	0.000*	− 0.087	− 0.026
Emotional neglect	0.022	0.231	0.012	− 0.006		0.050	0.031	0.326	0.027	0.004	0.059
Physical abuse	− 0.020	− 0.122	0.401	− 0.068		0.028	− 0.025	− 0.146	0.328	− 0.077	0.026
Physical neglect	0.030	0.193	0.193	− 0.190		0.079	0.055	0.343	0.031	0.005	0.015
Sexual abuse	0.036	0.231	0.017	0.007		0.065	0.040	0.330	0.007*	0.011	0.068
BMI	0.060	0.490	0.000*	0.034	0.085		0.050	0.427	0.000*	0.025	0.075
Smoker	0.147	0.140	0.181	− 0.070	0.363		0.178	0.169	0.112	− 0.43	0.399
*F*	4.743* (*df* = 7; 70)						5.817* (*df* = 7; 62)				
*R*^2^	0.345						0.425				
Corrected *R*^2^	0.272						0.352				

*p < 0.05 (for a global level of significance after correction for multiple testing via Bonferroni correction, equates a level of significance of *p* < 0.0071 for each variable)

### Treatment response in relation to ACE and inflammation

Finally, the multiple regression analysis for the treatment response after 2 weeks (*ΔMADRS_1)* showed no significant associations with none of the CTQ subscales nor the level of hsCRP at baseline under control of the BMI and the patient’s state concerning first/recurrent depressive episode. When setting the treatment response after 6 weeks (*ΔMADRS_2)* as dependent variable, the statistics showed no significant associations as well (see Table 5).

**Table 5:** Multiple regression analysis for *ΔMADRS* with CTQ, BMI, hsCRP and recurrent depression

	ΔMADRS_1					ΔMADRS_2				
	*β*	Stand *β*	*p*	95% CI		*β*	Stand β	*p*	95% CI	
Emotional abuse	− 0.442	− 0.304	0.151	− 1.051	0.167	− 0.567	− 0.324	0.167	− 1.382	0.247
Emotional neglect	0.519	0.350	0.061	− 0.024	1.062	0.487	0.284	0.165	− 0.208	1.182
Physical abuse	0.310	0.102	0.561	− 0.752	1.371	0.827	0.216	0.301	− 0.765	2.420
Physical neglect	0.006	0.003	0.089	− 0.945	0.0958	− 0.077	− 0.260	0.903	− 1.351	1.197
Sexual abuse	− 0.613	− 0.249	0.091	− 1.325	0.100	− 0.387	− 0.139	0.392	− 1.289	0.515
hsCRP	− 1.264	− 0.245	0.107	− 2.813	0.284	− 1.586	− 0.276	0.114	− 3.569	0.397
Recurrent Episode	3.642	− 0.245	0.107	− 2.813	8.611	2.563	0.116	0.452	− 4.250	9.376
BMI	− 0.026	− 0.14	0.927	− 0.585	0.534	− 0.003	− 0.001	0.993	− 0.717	0.711
*F*	(*df* = 8; 60)	1.626 (sig 0.14)				(*df* = 8;52)	0.882 (sig 0.539)			
*R*^2^	0.200					0.138				
Corrected *R*^2^	0.077					− 0.019				

*p* < 0.05; ΔMADRS_1 = MADRS (baseline) − MADRS (2 weeks), ΔMADRS_2 = MADRS (baseline) − MADRS (6 weeks)

*p* < 0.05 (for a global level of significance after correction for multiple testing via Bonferroni correction, equates a level of significance of *p* < 0.0063 for each variable)

## Discussion

### Summary

To our knowledge, this is the first study exploring the associations between specific forms of ACE, hsCRP and response to antidepressant treatment in patients diagnosed with uni- or bipolar depression. HsCRP levels in the overall study population, in the subgroups *early-improver* and *responder* did not significantly differ from the healthy control group. Multiple regression analysis showed that a higher BMI was significantly associated with higher levels of hsCRP at baseline, while the smoking status and the CTQ subscales did not. After 6 weeks of anti-depressant treatment with a significant decrease of the depressive symptoms and a significant increase of the hsCRP levels, the BMI and sexual abuse was associated with significant higher levels of hsCRP. While emotional abuse was associated with significant lower levels of hsCPR, the remaining CTQ subscales did not show any significant associations with the inflammation marker after correction for multiple testing. Concerning treatment response after 2 and 6 weeks, the statistical analyses showed no significant associations whether with the CTQ subscales nor with the hsCRP levels at baseline. The level of the inflammation marker at baseline did not significantly differ in the retrospectively built early-improver/non-early-improver or responder/non-responder state subgroups.

### ACE and hsCRP

Along with a growing amount of literature, the findings in our studies support the hypothesis of a long-lasting effect on the inflammation process following ACE [7, 15, 48]. The most suitable parameters for showing chronic inflammation after ACE remain unclear although hsCRP, IL-6 and TNF-alpha are being discussed as potential candidates [7, 57]. While the meta-analysis of Baumeister et al. [7] emphasises higher levels of TNF-α and IL-6 after physical and sexual abuse, they didn’t find this association for hsCRP. By contrast and along with our findings, more recent studies showed higher hsCRP levels after sexual abuse [1, 56] while other studies found similar associations after one or more ACE (without specifying the kind of trauma) [6, 48]. A study by Aas et al. [1] found higher levels of hsCRP in healthy controls as well as in a bipolar and schizophrenia mixed sample after childhood maltreatment but only before controlling for BMI.

With emotional abuse being associated with lower levels of hsCRP and sexual abuse showing higher hsCPR levels in the present population after the decrease of depressive symptoms, the question of why different types of ACE seem to have a different impact on inflammatory regulation processes becomes more and more urgent and should be considered in future research [7].

As stated before, the current literature is still heterogenous [7]. Our findings contribute to the heterogeneity of the available data with supporting the hypotheses of the long lasting effects of ACE on the human immune system. Especially the finding, that after a decrease of the depressive symptoms after 6 weeks, the associations of ACE and hsCRP became visible (for emotional abuse and sexual abuse), suggest both the influences of depression on hsCRP as well as the enduring impact of ACE. When looking at the CTQ subscales sexual abuse and emotional neglect at baseline as well as emotional and physical neglect after the treatment period, the multiple regression analysis showed significant positive correlations with hsCRP but only before correction for multiple testing. Here, more definite results would be eligible in further studies. Possible underlying molecular mechanisms are the subject of current research focusing on epigenetic regulation of gene expression (e.g., glucocorticoid receptor, FKBP5) and modifications of the hypothalamus-pituitary-adrenocortical axis [44, 50, 51].

### ACE and depression

In line with other studies, the prevalence of ACE varied from 11.8% for physical abuse to 61.8% for emotional neglect [59, 77]. ACE as relevant risk factor for affective disorders [13, 34, 36, 37, 40, 47, 75] was high in the present study population. Concerning response to antidepressant treatment Williams et al. [77] reported a significant prediction of response and remission to SSRI and SNRI treatment of sexual, physical and emotional abuse after 8 weeks of treatment with greater exposure leading to less response. The meta-analysis of Nanni et al. [58] also stated that patients with a history of childhood maltreatment benefit less from psychopharmacology, psychotherapy and combined therapy. Another study found that patients with multiple traumatisation or sexual abuse showed the worst response to psychotherapy treatment [66].

Considered as a very severe kind of traumatisation, the literature suggests that sexual abuse leaves the most lasting marks on the human body and soul [11, 16, 56, 66, 77]. As a possible conclusion, sexual abuse and multiple ACE could be considered as a relevant variable in treatment response. Nevertheless, we could not find any associations of specific forms of ACE and the response to anti-depressant treatment in this sample. The lack of a differentiate consideration of the treatment without a specific study protocol including pharmacological and non-pharmacological treatment implications could be an obvious explanation for this and is likely to be limiting for our findings. Nevertheless, specific treatment implications after ACE need further investigations. Besides psychopharmacology and psychotherapy, the treatment-setting itself could also play an important role. Emotionally neglected patients could maybe profit better from multimodal therapy settings; the face-to-face interpersonal experiences within the setting could be seen as an additional curative factor for people with unsatisfied attachment needs.

### hsCRP and depression

So far, the discussion about inflammatory biomarkers in depression has not come to an end [27, 29, 55, 69, 81]. Patients with depressive symptoms are considered to show higher levels of pro-inflammatory cytokines and acute-phase-proteins. The most investigated targets in the search for classifying and prognostic factors in current literature are IL-6, Il-1β, TNF-α and hsCRP [81]. The latter represents a common final pathway as the response to rising levels of IL-6 and TNF-α [15]. HsCRP is well established as a laboratory marker, is simple and cheap to measure and shows the most robust associations with ACE [15, 81]. Nevertheless, in our study, patients with uni- and bipolar depression did not show significantly higher levels of hsCRP compared to healthy controls as described in other studies [15, 27]. While the means of the hsCRP levels in the study population lay above the levels of the healthy controls, the differences did not reach statistical significance. By contrast, a recent study by Moraes et al. [56] did not find serum hsCRP levels to differ from healthy controls either. For bipolar-depression, there is less data providing information about levels of hsCRP, furthermore the available data is heterogenous [65]. Still Wysokinski et al. [80] could not find significant differences of CRP levels in a large sample of inpatients suffering from acute schizophrenia, bipolar mania or bipolar depression.

Concerning hsCRP itself, the literature suggests that the inflammatory response represented by CRP can be helpful identifying subgroups of depressed patients (e.g., atypical vs. typical depression) [45], but is less suitable for characterizing general depression [81]. Therefore, and considering the complexity of the inflammation process itself and the development of depressive symptoms, our findings support the statement that relying on a single inflammation marker is not expedient (for a further overview see e.g. Ref. [81]). The associations of depression and hsCRP were namely reported to be consistent but small (*r* = 0.05) [32]. In the field of low-grade inflammation, hsCRP appears to be very susceptible to confounders like e.g. age, sex, beginning infection, medication or the phase of a bipolar disorder [25, 32]. Concerning treatment response to antidepressant therapy however, inflammation markers are being discussed as having further clinical implications than for general characteristics of depression.

In contrast to our findings, recent literature identified higher levels of inflammation markers as being associated with treatment response [12, 71]. In the present study, we could not find any associations between hsCRP levels and treatment response. Possible explanations may lie in the missing distinction of the anti-depressants used within the open-label design. Several studies have identified levels of inflammation markers to be predictive for better response when treatment occurs with specific anti-inflammatory treatment [42, 64] or when differentiating into different subgroups of antidepressants [46, 73]. So, the response to antidepressant treatment is presumed to depend on baseline inflammation and the prescribed medication [5, 49]. Looking then at the trend of inflammation markers before and after anti-depressant treatment, Hiles et al. [31] found a normalisation of the measured biomarkers in most patients while another study found consistently high inflammation markers in patients who did not respond to antidepressant treatment [71].

While depressive symptoms significantly decreased over the study period, hsCRP levels did significantly increase during treatment. The MADRS and HAMD scores indicated only mild depressive symptoms after 6 weeks, whereas the BDI still indicated severe depression. This may be due to a subjective higher burden of the symptoms typical for depression in contrast to a more objective impression of a MADRS or HAMD rating. Conversely, the underlying immunological mechanisms and variations of inflammation markers in the treatment of depression are complex. Due to missing evidence, a substantiated statement concerning decrease or increase of hsCRP levels cannot be made so far [76]. To be able to make more precise statements concerning inflammation and specific treatment options in depression, further research is still needed.

### Limitations

The findings of the present study have to be considered under certain limitations. In consideration of the complex question of this study, the population was comparatively small and bigger cohort would have been preferable. Compared to unipolar depression, the development of bipolar depression is considered to have additionally underlying mechanisms and genetic influences. As there is evidence for higher rates of ACE as well as higher levels of hsCRP in both uni- and bipolar depression, the analysis here appear reasonable. Additionally, the percentage of bipolar patients in this sample was low. Nevertheless, when searching for explanations for the origin of those diseases, separate models for each diagnosis may be more revealing.

Current research is looking for more individual and more specific psychopharmacological and psychotherapy treatment regimens to gain better response rates when treating depression. In our study, treatment was unspecified and probably represents a therapy regime which is widespread in psychiatry departments and according to clinical experience. But concerning the results for treatment response, the lack of a more differentiated analysis of the types and dosages of the medication applied surely limits the findings of the study. When looking for prognostic factors and biomarkers for depression, a differentiated consideration of different treatment arms dividing into psychotherapy and/or varied drug regimens seems to be more conclusive to identify subgroups of depressed patients who might profit from a more personalised therapy. Besides the free choice of psychopharmacology during the study period, patients were also allowed to take any kind of medication without missing inclusion criteria at baseline. While current literature concerning the influences of anti-depressive medication on hsCRP is very heterogenous with recent articles reporting no significant influence on hsCRP, the missing effects of the prescribed medication at baseline on the inflammation marker needs further investigation. We excluded patients with a hsCRP level > 10 mg/dl. As especially obese women can show hsCRP levels higher than 10 mg/l as a result of chronic inflammation [35] this may lead to a reduction of the variance in the present sample. Nevertheless, along with the most other studies in this field we stick to the recommendations of the U. S. Centers for Disease Control and Prevention and the American Heart Association which sets the hsCRP threshold for acute inflammation at 10 mg/l. In numbers due to this limit, we excluded three patients whose hsCRP levels lay all above 20 mg/dl. Furthermore, ACE were assessed via self-report-questionnaires which are known for a tendency towards underreporting. Especially when participants are suffering from major depression, a mood-bias is probable.

## Conclusion

The present study emphasises an ongoing impact on inflammation regulation processes after specific forms of ACE. After correction for multiple testing, sexual abuse was associated with higher levels of hsCRP after the decline of the depressive symptoms in this uni- and bipolar sample. While emotional abused patients showed lower levels of hsCRP after the treatment period, we did not find any associations of ACE and hsCRP with the treatment response in the unipolar depressed subgroup in this study with its naturalistic design. As conducted in several other studies, in this context, we consider a more pre-defined study design with regard to medication and psychotherapy as more expedient. For future research and to reveal further clinical implications, the debate about biomarkers after ACE, the underlying mechanisms like e.g. epigenetic changes and biomarkers as a predictor for antidepressant treatment has to be continued, expanded and ideally brought together.

## Data Availability

Data are available from the Correspondence author.

## Code availability

Code is available from the Correspondence author; software application: SPSS 25.0
